# Enhancing social cognition in psychosis – the potential role of open dialogue

**DOI:** 10.1038/s41537-025-00608-y

**Published:** 2025-06-05

**Authors:** Maria Gariup, Tomi Bergström, Katharina Saliger, Justin M. Palanci, Robert O. Cotes, Joseph B. Stoklosa, Jaakko Seikkula

**Affiliations:** 1 Child and Adolescent Psychiatric Center Glostrup, Psychosis Reference Unit - Opus Young Program, 2600 Glostrup, Denmark; 2 Department of Psychology, University of Jyväskylä, Jyväskylä, Finland; 3 Hospital of Interlaken, Psychiatry Interlaken, Interlaken, 3800 Switzerland; 4 Department of Psychiatry and Behavioural Sciences, Emory University School of Medicine, 10 Park Place SE, Suite 620, Atlanta, GA 30303 USA; 5 Harvard Medical School, Department of Psychiatry, McLean Hospital, 115 Mill St, Belmont, MA 02474 USA

**Keywords:** Psychology, Emotion, Psychosis, Schizophrenia

## Abstract

Research in psychotic conditions increasingly focuses on improving functional outcomes. While antipsychotic treatments can alleviate positive symptoms in some patients, they have limited impact on psychosocial function. Social cognition (SC) has emerged as a promising target for intervention, with multiple new approaches designed to enhance SC in psychotic conditions. However, the generalisability of these interventions to real-world functioning remains uncertain. We propose that the Finnish Open Dialogue (OD) approach may offer unique advantages for improving SC in individuals with psychosis and their families and suggest this as an important direction for future research. This commentary aims to bridge perspectives from SC research and OD practice, proposing avenues for integration and future research.

## Overview

Research increasingly focuses on improving functional outcomes in psychotic conditions. While antipsychotics can alleviate positive symptoms in some patients^1^, their impact on psychosocial function is limited^2,3^. Social cognition (SC), the cognitive processes underlying interpersonal interactions, has emerged as a promising intervention area, as impairments in SC correlate with poorer functional outcomes. While structured SC training programs show promise, their generalisability to real-world functioning remains uncertain^4,5^.

The Open Dialogue approach to psychosis^6^, offers a dialogical, network-oriented approach that may uniquely enhance SC in subjects with psychotic conditions and their families by addressing relational and contextual dynamics, complementing existing interventions.

Here, we comment on the core principles of OD and their potential contribution to SC improvements, acknowledging that empirical studies are needed to assess these mechanisms.

This commentary bridges perspectives from SC research and OD practice, proposing avenues for integration and future research.

## Social cognition

Social cognition (SC) refers to how individuals perceive, interpret, and evaluate social behaviours^7^, or simply put, “it is people thinking about people”^8^. It encompasses mental processes vital for adaptive social interactions. Research in psychotic conditions has typically focused on four main domains: emotion perception and processing, mentalizing, social perception and social knowledge, and attributional bias^4,9–12^.

*Emotion perception and processing (EPP)* involves perceiving and using emotions adaptively. *Mentalizing* or *Theory of Mind (ToM)* is the ability to understand others’ intentions and emotions, including detecting sincerity, deception or sarcasm. *Social perception (SP)* assesses the ability to identify social roles and rules from non-verbal cues, as voice intonation, mimics, proxemics. *Attributional bias (AB)* refers to how different individuals infer causes for events, such as attributing - or not - hostile intentions to others in ambiguous social situations^4^.

### Social cognition abilities in psychotic conditions

Substantial evidence indicates that emotion processing, mentalizing, and social perception are impaired in individuals with psychotic conditions, while evidence for attributional bias is less conclusive^4,10,13^. These impairments appear to be core features, present in chronic and first-episode patients, independent of symptoms or medication, and observable at some degree in unaffected relatives and in high-risk, prodromal samples^14–16^.

Studies suggest that SC abilities are associated with functional outcomes both pre-morbidly, cross-sectionally, and longitudinally, with moderate effects^17–20^. A recent meta-analysis found that SC measures had the strongest association with psychosocial functioning among all cognitive variables, explaining more variation than intelligence measures (as IQ, working memory, executive functions)^18^.

Interventions enhancing SC may therefore significantly improve outcomes^17,18,21^ and several approaches have emerged.

### Social cognition training in psychotic conditions

SC emerged at the intersection of cognitive, developmental and social psychology, and neuroscience, integrating diverse perspectives. It draws from social learning theory^22^, which emphasizes observational learning and modelling; attribution theory^23,24^, which explores how individuals infer causes for others’ behaviours; Theory of Mind frameworks^25–27^, which focus on mindreading and perspective-taking; and neuroscientific insights into social brain networks^28,29^. Attachment theory^30,31^ highlights how early caregiver relationships shape mentalizing and emotion regulation —core aspects of SC.

Recent perspectives, including psychological constructionism, interactionism, and embodiment theories, emphasize the relational and experiential nature of SC, proposing that interpersonal dynamics and bodily experiences shape SC development. Several authors suggest integrative perspectives^32–38^.

These theoretical frameworks inform the different SC training (SCT) interventions (for overviews see refs. ^39,40^).

Typical training practices include psychoeducation, use of visual media as pictures or short film clips to recognize emotions^41^, infer mental states^42^ or interpret social cues^11,43^, role-play scenarios, and cognitive restructuring^44^. SCT programs can be “targeted”, addressing one SC domain, or “broad-based”, addressing several SC domains. E.g., Training of Affect Recognition (TAR)^45^ addresses EPP, Metacognitive Training (MCT)^46^ primarily ToM, and Social Cognition and Interaction Training (SCIT) integrates social learning and cognitive restructuring to improve EPP, SP, and AB^47^. Some interventions combine SCT with neurocognitive training, as in Cognitive Enhancement Therapy (CET)^48^, while others employ virtual reality (VR) to enhance ecological validity^5,49–51^ or mindfulness-based trainings to improve nonverbal social attunement^52^.

While SCTs demonstrate efficacy in improving EPP, ToM and SP, meta-analyses indicate no significant impact on AB or symptom severity^5,11,53^, and mixed results on functional outcomes^5,40,53,54^, with the latest meta-analysis showing no improvements in schizophrenia^53^.

One of the main challenges of SCTs is their generalizability to real-world social interactions, and a possible “train to the test” effect, with unclear improvements in everyday social functioning^4,5,53,55^. Efforts to increase ecological validity with VR-based trainings^5,53^ have so far produced negative results, suggesting that emotional expression in virtual environments may be inadequate^51^. Additional barriers include high decline rates due to social anxiety and privacy concerns^40^.

Some scholars posit that SCTs may need critical re-evaluation, proposing more comprehensive approaches and integration with other interventions^5,51,56^.

In this context, OD may offer a relational, integrative approach that situates SC development within authentic, interactive settings, complementing existing interventions and potentially enhancing SC across individuals, families, and professional networks.

## An overview of open dialogue

Open Dialogue (OD) is a family-centred, team-based, need-adapted approach originally developed in Western Lapland, Finland, for psychotic crises^57^. It provides rapid, low-threshold support based on seven core principles: immediate help, a social network perspective, flexibility and mobility, responsibility, psychological continuity, tolerance of uncertainty, and dialogue^58^. Individuals can contact the service directly, and network meetings are promptly arranged, involving all relevant persons at convenient locations. Treatment is tailored to individual needs, and may incorporate psychotherapy, trauma treatment, and social support. The initial treatment team remains responsible throughout, ensuring continuity of care. Providing prompt support and security enables tolerance of uncertainty, allowing time before committing to long-term diagnoses or treatments. All treatment decisions are made transparently and collectively^57,59^.

Central to OD is promoting dialogue among all participants: professionals facilitate open conversations where every perspective is respected, and share their thoughts and feelings aloud in “reflective conversations”, inviting the network to respond^60,61^. This collaborative process fosters mutual understanding and creates a supportive environment^62^.

### OD - outcomes

OD has shown positive outcomes in symptom, functional recovery and medication use compared to standard care in Finland^6^, and these findings were sustained at the 19-year follow-up^62^. Over time in the region, the duration of untreated psychosis decreased to half month, and the incidence of schizophrenia diagnoses dropped significantly^6^. Similar outcomes have been observed in adolescents^63^. Internationally, OD led to various implementations and practices^64^, along with a forthcoming randomized controlled trial in the UK^65^.

Recent developments have introduced peer participation in Peer-Supported OD (POD)^66^, aligning with recovery-oriented models^67,68^. Throughout this commentary, the term ‘professionals’ encompasses both formal therapists and peer workers.

## Open dialogue and social cognition

OD shares various elements with other first-episode programs^69^, yet certain specific aspects may enhance SC abilities uniquely: mechanisms as learning by modelling, perspective-taking, emotion recognition and regulation, ecological validity, a relational perspective, body attunement permeate the approach. This section outlines how OD may influence each SC domain, and the theoretical background supporting that.

Relevant across all SC domains is Vygovtsky’s concept of “zone of proximal development” (ZPD)^70^, frequently referenced in OD literature. ZPD describes the metaphorical space between an individual’s current abilities and their potential development, achievable through interaction with more experienced partners, in a process of mutual cooperation, where the more experienced participant continuously adjusts their approach to support the other’s growth^71,72^.

### Emotion perception and processing - EPP

The focus on emotions is central in OD, that supports emotion expression and aims at “creating a shared emotional experience”, that would allow relational healing^72,73^. Professionals pay close attention to subtle emotional shifts, and model emotional awareness by verbalizing their own emotions and bodily sensations, often echoing those of others in the conversation. Professionals proceed calmly and show that it is possible to talk about difficult experiences and to sustain difficult emotions, fostering feelings of safety in network members^72–74^.

Explicit emotion labelling is thought to enhance EPP, as accessible emotion concepts would help individuals make sense of affective experiences^32,75,76^. Moreover, labelling emotions would have a self-regulatory effect^75,77,78^, further facilitating social understanding^79^.

Social experience is considered crucial for developing emotion recognition^80^, and emotional learning thought to thrive in safe relationships^79,81,82^ and in multimodal interactions with familiar individuals^83^.

OD meetings often result in collective decisions and actions, providing participants with direct learning of how emotions can be harnessed to bring about meaningful change^84^, another central facet of EPP^85,86^.

By integrating these processes, OD may enable all participants to refine their ability to identify and interpret emotions in dynamic and complex exchanges.

### Mentalizing - ToM

Through dialogues, OD participants may gain insight into both their own and others perspectives^62,72^. Reflective conversations model self-reflection and perspective-taking, scaffolding the process for the network^72,74^. Team members adapt to the network language, and work to construct a new “shared language”, where each participant can express and make sense of their experiences^72^. Through this meaning-making process, one’s personal history can be integrated into a meaningful narrative, fostering autobiographical coherence^87^.

Metacognitive and mentalization-based theories emphasize that mentalizing capacity is shaped dynamically through interpersonal dialogue and shared meaning-making^88,89^. Interactive theories suggest that real-time social interaction leads to a deeper understanding of others’ mental states than passive observation^34,90^, and autobiographical knowledge is linked to mentalizing capacities^91–94^.

Indirect empirical evidence from affine approaches also supports OD’s potential to enhance mentalizing. Mentalization-based treatment (MBT) can improve ToM in psychotic conditions^95–97^, and Family-Based Mentalization Therapy (FBMT) has improved mentalizing and family communication in high conflict families of adolescents^98–100^. While MBT/FMBT and OD differ structurally, they share non-directive therapeutic engagement, dialogical exploration, and a relational focus, suggesting similar potential benefits.

The emotional safety is also critical for sustaining a mentalizing stance, as it fosters openness to alternative interpretations of social interactions^82^.

OD may thus offer a naturalistic, interactive setting, that may enhance flexible and adaptive mentalizing in all participants.

### Social perception and social knowledge (SP)

OD acknowledges non-verbal communication as a crucial component of dialogue^101^. Professional express their physical and emotional responses to the environment, modelling attunement to subtle social signals^73,74^. Mindfulness practices have been incorporated into OD trainings to support this^64^. This could encourage participants to detect and interpret intonation, gestures, posture, and proxemics, and deepen their understanding of implicit social dynamics, in line with embodiment theories, suggesting that aspects of social cognition are grounded in sensorimotor and bodily experiences^37^.

Interpreting social cues within context is fundamental to SP^102^. The collaborative meaning-making process engages all participants in constructing a coherent understanding of situations^62,87^, and the collective decision-making process can provide direct opportunities for social negotiation, collaboration, and conflict resolution, giving insight about social roles and relational dynamics. This is consistent with interactionist perspectives, positing that both interacting socially^38,90^ and engaging in joint action with others^33^ would give access to more information about the behaviour of one’s partners, than their simple observation.

Improvements in SP are linked to enhanced functional outcomes, prompting calls for SP-focused interventions^11,39,40^. OD may provide an ecologically valid approach by embedding SP learning in dynamic, real-world interactions.

### Attributional bias

Individuals with psychotic conditions may tend to perceiving hostility in ambiguous social situations^13^, increasing the risk of delusional beliefs, anxiety, agitation, and depressive symptoms^103^: transparency in OD discussions and decisions may dispel assumptions and misunderstandings^62^, promoting more balanced attributions. OD’s personalized approach may also enhance trust in the system^104^, thus promoting epistemic trust and openness to new information that may arise^105,106^.

Individuals in psychotic crises can also often feel lonely and isolated, which can increase pessimism and negative biases^107^. OD collaborative problem-solving and collective engagement in common goals may enhance trust and belonging, and peer involvement further contribute to normalization, stigma reduction and hope fostering^64,108,109^, potentially supporting more benign attributional styles^107^.

Constructing coherent autobiographical narratives may also help individuals reinterpret past and current experiences through a relational lens, fostering self-other differentiation and reducing extreme attributions of blame or hostility^87^.

OD may offer a relational pathway to cognitive flexibility and social belonging by fostering trust and mutual understanding within real social-relational contexts.

### Summary on SC and OD

OD holds the potential to enhance each subdomain of social cognition.

Interventions targeting multiple domains simultaneously appear most beneficial^5,55^. Individuals with psychotic experiences are not a homogeneous group: their social cognition is linked to unique life contexts and relationships, highlighting the need for flexible approaches^110^.

## Addressing compatibility and contextual considerations

OD’s relational stance contrasts with individualist paradigms underlying SC research. However, these perspectives may be complementary. Rather than focusing solely on the individual, OD addresses relational dynamics, potentially enhancing SC across entire networks. This may create a mutually reinforcing process that improves communication patterns and reduces distress within the broader system. Positive results of MBTs applied to families^100,111^, align with OD’s emphasis on shared responsibility for change.

Given that cognition alone does not fully account for social functioning^18,112^, researchers advocate for combining SC training with broader psychosocial interventions^53,56^. OD integrative and network-based approach may already offer such comprehensive support^113^.

Psychosis research increasingly incorporates “personal recovery”, alongside symptom and functional outcomes^112,114^. OD aligns with recovery-informed interventions^68,114^, potentially expanding the spectrum of its meaningful outcomes^115,116^.

The global applicability of OD requires careful evaluation. In Finland, OD allows direct service access without referrals or diagnoses, a model rarely replicable elsewhere. Adaptations often require structural adjustments^64^. Also, while OD has gained international clinical interest, empirical research outside Finland remains limited, highlighting the need for further study^64,65^.

## Conclusions

OD holds promise to enhance SC in psychosis through its foundation in dialogue, transparency, and mutual respect: “Dialogue increases reflexivity”^73^.

Its relational focus suggests it as a complementary approach to existing interventions: the inclusion of family members may improve the entire network’s social cognition, enhancing communication and reducing distress. Family participation may also address challenges as recruitment difficulties due to social anxiety or privacy issues^40^.

The theoretical nature of these claims should be acknowledged. Future studies could assess SC outcomes pre- and post-OD intervention or compare OD with standard SCT programs. Examining SC changes across the entire network appears relevant. Additionally, exploring OD’s applicability in populations with primary SC deficits, such as autism spectrum conditions, could expand its relevance beyond psychosis.

These reflections are particularly relevant in the context of the global mental health crisis, where experts advocate for a transformation of mental health systems, by addressing social environment, adopting rights-based approaches, involving peers, implementing psychosocial interventions, and ensuring continuity of care^117^. OD is well-aligned with such principles, suggesting its potential as one model for future mental health practices.

## Data Availability

This commentary is based on a review of existing literature and does not include new data. All referenced studies and sources are publicly available in the cited works.

## References

[1] Leucht, S. et al. Comparative efficacy and tolerability of 15 antipsychotic drugs in schizophrenia: A multiple-treatments meta-analysis. *Lancet* **382**, 951–962 (2013). 23810019 10.1016/S0140-6736(13)60733-3

[2] McGorry, P. D., Killackey, E. & Yung, A. Early intervention in psychosis: concepts, evidence and future directions. *World Psychiatry* **7**, 148–156 (2008). 18836582 10.1002/j.2051-5545.2008.tb00182.x PMC2559918

[3] Swartz, M. S. et al. Effects of antipsychotic medications on psychosocial functioning in patients with chronic schizophrenia: findings from the NIMH CATIE study. *Am. J. Psychiatry* **164**, 428–436 (2007). 17329467 10.1176/ajp.2007.164.3.428

[4] Green, M. F., Horan, W. P. & Lee, J. Nonsocial and social cognition in schizophrenia: current evidence and future directions. *World Psychiatry* **18**, 146–161 (2019). 31059632 10.1002/wps.20624 PMC6502429

[5] Nijman, S. A., Veling, W., van der Stouwe, E. C. D. & Pijnenborg, G. H. M. Social cognition training for people with a psychotic disorder: A network meta-analysis. *Schizophr. Bull.* **46**, 1086–1103 (2020). 32162658 10.1093/schbul/sbaa023 PMC7505203

[6] Seikkula, J., Alakare, B. & Aaltonen, J. The comprehensive open-dialogue approach in western lapland: II. Long-term stability of acute psychosis outcomes in advanced community care. *Psychosis* **3**, 192–204 (2011).

[7] American Psychological Association. (n.d.). Social Cognition. In APA dictionary of psychology. Retrieved October 20, 2024. https://dictionary.apa.org/social-cognition (2024).

[8] Green, M. F., Olivier, B., Crawley, J. N., Penn, D. L. & Silverstein, S. Social cognition in schizophrenia: Recommendations from the Measurement and Treatment Research to Improve Cognition in Schizophrenia New Approaches Conference. *Schizophr. Bull.* **31**, 882–887 (2005). 16135561 10.1093/schbul/sbi049

[9] Green, M. F. et al. Social cognition in schizophrenia: an NIMH workshop on definitions, assessment, and research opportunities. *Schizophr. Bull.* **34**, 1211–1220 (2008). 18184635 10.1093/schbul/sbm145 PMC2632490

[10] Savla, G. N., Vella, L., Armstrong, C. C., Penn, D. L. & Twamley, E. W. Deficits in domains of social cognition in schizophrenia: a meta-analysis of the empirical evidence. *Schizophr. Bull.* **39**, 979–992 (2013). 22949733 10.1093/schbul/sbs080 PMC3756768

[11] Kurtz, M. M., Gagen, E., Rocha, N. B. F., Machado, S. & Penn, D. L. Comprehensive treatments for social cognitive deficits in schizophrenia: A critical review and effect-size analysis of controlled studies. *Clin. Psychol. Rev.* **43**, 80–89 (2016). 26437567 10.1016/j.cpr.2015.09.003

[12] Pinkham, A. E. et al. The social cognition psychometric evaluation study: Results of the expert survey and RAND Panel. *Schizophr. Bull.* **40**, 813–823 (2014). 23728248 10.1093/schbul/sbt081 PMC4059426

[13] Healey, K. M., Bartholomeusz, C. F. & Penn, D. L. Deficits in social cognition in first episode psychosis: A review of the literature. *Clin. Psychol. Rev.* **50**, 108–137 (2016). 27771557 10.1016/j.cpr.2016.10.001

[14] Lee, T. Y., Hong, S., Bin, Shin, N. Y. & Kwon, J. S. Social cognitive functioning in prodromal psychosis: A meta-analysis. *Schizophr. Res.* **164**, 28–34 (2015). 25749019 10.1016/j.schres.2015.02.008

[15] McCleery, A., Horan, W. P. & Green, M. F. Social Cognition during the Early Phase of Schizophrenia. *Soc. Cogn. Metacognition Schizophr*. 49–67, 10.1016/b978-0-12-405172-0.00003-x (2014).

[16] Glenthøj, L. B. et al. Social cognition in patients at ultra-high risk for psychosis: What is the relation to social skills and functioning? *Schizophr. Res. Cogn.* **5**, 21–27 (2016). 28740813 10.1016/j.scog.2016.06.004 PMC5514303

[17] Halverson, T. F. et al. Pathways to functional outcomes in schizophrenia spectrum disorders: Meta-analysis of social cognitive and neurocognitive predictors. *Neurosci. Biobehav. Rev.* **105**, 212–219 (2019). 31415864 10.1016/j.neubiorev.2019.07.020

[18] Cowman, M. et al. Cognitive Predictors of Social and Occupational Functioning in Early Psychosis: A Systematic Review and Meta-analysis of Cross-Sectional and Longitudinal Data. *Schizophr. Bull.* **47**, 1243–1253 (2021). 33761534 10.1093/schbul/sbab033 PMC8379554

[19] Punsoda-Puche, P., Barajas, A., Mamano-Grande, M., Jiménez-Lafuente, A. & Ochoa, S. Relationship between social cognition and premorbid adjustment in psychosis: a systematic review. *Schizophrenia* **10**, 36 (2024). 10.1038/s41537-023-00428-y PMC10942991 38491028

[20] Fett, A. K. J. et al. The relationship between neurocognition and social cognition with functional outcomes in schizophrenia: A meta-analysis. *Neurosci. Biobehav. Rev.* **35**, 573–588 (2011). 20620163 10.1016/j.neubiorev.2010.07.001

[21] Peña, J. et al. Combining social cognitive treatment, cognitive remediation, and functional skills training in schizophrenia: A randomized controlled trial. *npj Schizophr*. **2**, 16037 (2016). 10.1038/npjschz.2016.37 PMC5102241 27868083

[22] Bandura, A. *Social Foundations of Thought and Action: A Social Cognitive Theory*. vol. 16 (Engelwood Cliffs. Prentice Hall., 1986).

[23] Heider, F. *The psychology of interpersonal relations*. *The psychology of interpersonal relations* (John Wiley & Sons Inc, 1958). 10.1037/10628-000.

[24] Weiner, B. An attributional theory of achievement motivation and emotion. *Psychological Rev.* **92**, 548–573 (1985). 3903815

[25] Premack, D. & Woodruff, G. Does the chimpanzee have a theory of mind? *Behav. Brain Sci.* **1**, 515–526 (1978).

[26] Frith, C. D. & Frith, U. The Neural Basis of Mentalizing. *Neuron* **50**, 531–534 (2006). 16701204 10.1016/j.neuron.2006.05.001

[27] Baron-Cohen, S., Leslie, A. M. & Frith, U. Does the autistic child have a ‘theory of mind’. *Cognition* **21**, 37–46 (1985). 2934210 10.1016/0010-0277(85)90022-8

[28] Adolphs, R. The neurobiology of social cognition. *Curr. Opin. Neurobiol.* **11**, 231–239 (2001). 11301245 10.1016/s0959-4388(00)00202-6

[29] Ekman, P. An Argument for Basic Emotions. *Cogn. Emot.* **6**, 169–200 (1992).

[30] Bowlby, J. *Attachment and loss: vol. 1. attachment*. (Basic Books, 1969).

[31] Fonagy, P. & Bateman, A. W. Adversity, attachment, and mentalizing. *Compr. Psychiatry* **64**, 59–66 (2016). 26654293 10.1016/j.comppsych.2015.11.006

[32] Lindquist, K. A., MacCormack, J. K. & Shablack, H. The role of language in emotion: predictions from psychological constructionism. *Front. Psychol.* **6**, 444 (2015). 25926809 10.3389/fpsyg.2015.00444 PMC4396134

[33] Gallotti, M. & Frith, C. D. Social cognition in the we-mode. *Trends Cogn. Sci.* **17**, 160–165 (2013). 23499335 10.1016/j.tics.2013.02.002

[34] Michael, J. Interactionism and mindreading. *Rev. Philos. Psychol.* **2**, 559–578 (2011).

[35] Bohl, V. & van den Bos, W. Towards an integrative account of social cognition: Marrying theory of mind and interactionism to study the interplay of Type 1 and Type 2 processes. *Front. Hum. Neurosci*. 10.3389/FNHUM.2012.00274 (2012). 10.3389/fnhum.2012.00274 PMC3468956 23087631

[36] Lux, V. et al. A Developmental Framework for Embodiment Research: The Next Step Toward Integrating Concepts and Methods. *Front. Syst. Neurosci.* **15**, 1–22 (2021). 10.3389/fnsys.2021.672740 PMC8360894 34393730

[37] Uithol, S. & Gallese, V. The role of the body in social cognition. *Wiley Interdiscip. Rev. Cogn. Sci.* **6**, 453–460 (2015). 26267834 10.1002/wcs.1357

[38] Schilbach, L. et al. Toward a second-person neuroscience. *Behav. Brain Sci.* **36**, 393–414 (2013). 23883742 10.1017/S0140525X12000660

[39] Grant, N., Lawrence, M., Preti, A., Wykes, T. & Cella, M. Social cognition interventions for people with schizophrenia: a systematic review focussing on methodological quality and intervention modality. *Clin. Psychol. Rev.* **56**, 55–64 (2017). 28688282 10.1016/j.cpr.2017.06.001

[40] Tang, J. M. Y. et al. Social cognition interventions for patients with first-episode psychosis: A scoping review. *Psychiatry Res.* **342**, 116191 (2024). 39303555 10.1016/j.psychres.2024.116191

[41] Roberts, D. L., Penn, D. L. & Combs, D. R. *Social cognition and interaction training (SCIT): group psychotherapy for schizophrenia and other psychotic disorders clinician guide*. 231 (Oxford University Press, 2016).

[42] Green, M. F., Horan, W. P. & Lee, J. Social cognition in schizophrenia. *Nat. Rev. Neurosci*. **16**, 620–631 (2015). 10.1038/nrn4005 26373471

[43] Horan, W. P. et al. Efficacy and specificity of Social Cognitive Skills Training for outpatients with psychotic disorders. *J. Psychiatr. Res.* **45**, 1113–1122 (2011). 21377168 10.1016/j.jpsychires.2011.01.015 PMC4064828

[44] Moritz, S. et al. Further evidence for the efficacy of a metacognitive group training in schizophrenia. *Behav. Res. Ther.* **49**, 151–157 (2011). 21276962 10.1016/j.brat.2010.11.010

[45] Wölwer, W. et al. Remediation of impairments in facial affect recognition in schizophrenia: Efficacy and specificity of a new training program. *Schizophr. Res.* **80**, 295–303 (2005). 16125367 10.1016/j.schres.2005.07.018

[46] Moritz, S., Vitzthum, F., Randjbar, S., Veckenstedt, R. & Woodward, T. S. Detecting and defusing cognitive traps: metacognitive intervention in schizophrenia. *Curr. Opin. Psychiatry* **23**, 561–569 (2010). 20683332 10.1097/YCO.0b013e32833d16a8

[47] Penn, D. L., Roberts, D. L., Combs, D. & Sterne, A. Best practices: The development of the Social Cognition and Interaction Training program for schizophrenia spectrum disorders - PubMed. *Psychiatr. Serv.* **58**, 449–451 (2007). 17412842 10.1176/ps.2007.58.4.449

[48] Hogarty, G. E., Hogarty, G. E. & Greenwald, D. P. *Cognitive enhancement therapy: The training manual*. (University of Pittsburgh Medical Center, 2006).

[49] Bronfenbrenner, U. *Experiments by Nature and Design* (Harvard University Press, 1979), 10.4159/9780674028845.

[50] Barker, R. G. *Ecological Psychology: Concepts and Methods for Studying the Environment of Human Behaviour* (Stanford University Press, 1968).

[51] Nijman, S. A. et al. Dynamic Interactive Social Cognition Training in Virtual Reality (DiSCoVR) versus Virtual Reality Relaxation (VRelax) for People with a Psychotic Disorder: A Single-Blind Multicenter Randomized Controlled Trial. *Schizophr. Bull.* **49**, 518–530 (2023). 36413388 10.1093/schbul/sbac166 PMC10016415

[52] Mediavilla, R. et al. Mindfulness-based social cognition training (SocialMIND) versus psychoeducational multicomponent intervention for people with a first episode of psychosis: a study protocol for a randomised controlled trial. *BMC Psychiatry* **19**, 233 (2019). 31357965 10.1186/s12888-019-2206-4 PMC6664759

[53] Yeo, H., Yoon, S., Lee, J., Kurtz, M. M. & Choi, K. A meta‐analysis of the effects of social‐cognitive training in schizophrenia: The role of treatment characteristics and study quality. *Br. J. Clin. Psychol.* **61**, 37–57 (2021). 34291465 10.1111/bjc.12320

[54] Bordon, N., O’Rourke, S. & Hutton, P. The feasibility and clinical benefits of improving facial affect recognition impairments in schizophrenia: Systematic review and meta-analysis. *Schizophr. Res.* **188**, 3–12 (2017). 28095998 10.1016/j.schres.2017.01.014

[55] Rocha, N. B. F. & Queirós, C. Metacognitive and social cognition training (MSCT) in schizophrenia: a preliminary efficacy study. *Schizophr. Res.* **150**, 64–68 (2013). 23962827 10.1016/j.schres.2013.07.057

[56] Horan, W. P. & Green, M. F. Treatment of social cognition in schizophrenia: Current status and future directions. *Schizophr. Res.* **203**, 3–11 (2019). 28712968 10.1016/j.schres.2017.07.013

[57] Seikkula, J. & Olson, M. E. The open dialogue approach to acute psychosis: Its poetics and micropolitics. *Fam. Process* **42**, 403–418 (2003). 14606203 10.1111/j.1545-5300.2003.00403.x

[58] Seikkula, J. *et al*. Treating psychosis by means of open dialogue. In *The reflecting team in action: collaborative practice in family therapy* (ed. Friedman, S.) (The Guilford Press., 1995).

[59] Bergström, T., Seikkula, J., Köngäs-Saviaro, P., Taskila, J. J. & Aaltonen, J. Need adapted use of medication in the open dialogue approach for psychosis: a descriptive longitudinal cohort study. *Psychosis* **15**, 134–144 (2023).

[60] Olson, M., Seikkula, J., Ziedonis, D., Olson, J. & Ziedonis, D. The Key Elements Of Dialogic Practice In Open Dialogue: Fidelity Criteria. University of Massachusetts Medical School. Retrieved from https://www.umassmed.edu/globalassets/psychiatry/open-dialogue/keyelementsv1.109022014.pdf.

[61] Sidis, A. E., Moore, A. R., Pickard, J. & Deane, F. P. Always opening and never closing’: How dialogical therapists understand and create reflective conversations in network meetings. *Front. Psychol.* **13**, 992785 (2022). 36275250 10.3389/fpsyg.2022.992785 PMC9580692

[62] Bergström, T. et al. The family-oriented open dialogue approach in the treatment of first-episode psychosis: Nineteen–year outcomes. *Psychiatry Res* **270**, 168–175 (2018). 30253321 10.1016/j.psychres.2018.09.039

[63] Bergström, T. et al. The 10-year treatment outcome of open dialogue-based psychiatric services for adolescents: A nationwide longitudinal register-based study. *Early Interv. Psychiatry* **16**, 1368–1375 (2022). 35332989 10.1111/eip.13286 PMC10078679

[64] Pocobello, R. et al. Open Dialogue services around the world: a scoping survey exploring organizational characteristics in the implementation of the Open Dialogue approach in mental health services. *Front. Psychol.* **14**, 1241936 (2023). 38023059 10.3389/fpsyg.2023.1241936 PMC10668593

[65] Pilling, S. et al. Open Dialogue compared to treatment as usual for adults experiencing a mental health crisis: Protocol for the ODDESSI multi-site cluster randomised controlled trial. *Contemp. Clin. Trials* **113**, 106664 (2022). 10.1016/j.cct.2021.106664 PMC8844585 34958932

[66] Kinane, C., Osborne, J., Ishaq, Y., Colman, M. & MacInnes, D. Peer supported Open Dialogue in the National Health Service: implementing and evaluating a new approach to Mental Health Care. *BMC Psychiatry* **22**, 138 (2022). 35193551 10.1186/s12888-022-03731-7 PMC8862567

[67] Slade, M. et al. Uses and abuses of recovery: Implementing recovery-oriented practices in mental health systems. *World Psychiatry* **13**, 12–20 (2014). 24497237 10.1002/wps.20084 PMC3918008

[68] Winsper, C., Crawford-Docherty, A., Weich, S., Fenton, S.-J. J. & Singh, S. P. How do recovery-oriented interventions contribute to personal mental health recovery? A systematic review and logic model. *Clin. Psychol. Rev.* **76**, 101815 (2020). 32062302 10.1016/j.cpr.2020.101815

[69] Puntis, S. et al. Specialised early intervention teams for recent-onset psychosis. *Cochrane Database Syst. Rev*. **2020**, CD013288 (2020). 10.1002/14651858.CD013288.pub2 PMC8092671 33135811

[70] Vygotsky, L. *Thought and language. Thought and language* (MIT Press, 1962), 10.1037/11193-000.

[71] Leiman, M. Dialogical Sequence Analysis and the Zone of Proximal Development as Conceptual Enhancements to the Assimilation Model: The Case of Jan Revisited. *Psychother. Res.* **11**, 311–330 (2001).

[72] Seikkula, J. & Trimble, D. Healing elements of therapeutic conversation: Dialogue as an embodiment of love. *Fam. Process* **44**, 461–475 (2005). 16433289 10.1111/j.1545-5300.2005.00072.x

[73] Seikkula, J. Il dialogo guarisce. Ecco perché. Pensa Multimedia (2023).

[74] Andersen, T. Reflecting processes; acts of informing and forming: You can borrow my eyes, but you must not take them away from me! In: *The reflecting team in action: Collaborative practice in family therapy* (ed. Friedman, S.) 11–37 (Guilford Press, 1995).

[75] Brooks, J. A. et al. The role of language in the experience and perception of emotion: a neuroimaging meta-analysis. *Soc. Cogn. Affect. Neurosci.* **12**, 169–183 (2017). 27539864 10.1093/scan/nsw121 PMC5390741

[76] Nook, E. C., Lindquist, K. A. & Zaki, J. A new look at emotion perception: Concepts speed and shape facial emotion recognition. *Emotion* **15**, 569–578 (2015). 25938612 10.1037/a0039166

[77] Lieberman, M. D. Why symbolic processing of affect can disrupt negative affect: Social cognitive and affective neuroscience investigations. In: *Social neuroscience: Toward understanding the underpinnings of the social mind*, 188–209 (Oxford University Press, 2011). 10.1093/acprof:oso/9780195316872.003.0013.

[78] Torre, J. B. & Lieberman, M. D. Putting Feelings Into Words: Affect Labeling as Implicit Emotion Regulation. *Emot. Rev.* **10**, 116–124 (2018).

[79] Porges, S. W. The polyvagal theory: Phylogenetic substrates of a social nervous system. *Int. J. Psychophysiol.* **42**, 123–146 (2001). 11587772 10.1016/s0167-8760(01)00162-3

[80] Harms, M. B., Martin, A. & Wallace, G. L. Facial Emotion Recognition in Autism Spectrum Disorders: A Review of Behavioral and Neuroimaging Studies. *Neuropsychol. Rev.* **20**, 290–322 (2010). 20809200 10.1007/s11065-010-9138-6

[81] Baker, J. T. et al. Functional connectomics of affective and psychotic pathology. *Proc. Natl Acad. Sci. USA* **116**, 9050–9059 (2019). 30988201 10.1073/pnas.1820780116 PMC6500110

[82] Holmes, J. & Slade, A. The neuroscience of attachment: implications for psychological therapies. *Br. J. Psychiatry* **214**, 318–319 (2019). 31088592 10.1192/bjp.2019.7

[83] Grossmann, T. The development of emotion perception in face and voice during infancy. *Restor. Neurol. Neurosci.* **28**, 219–236 (2010). 20404410 10.3233/RNN-2010-0499

[84] Lerner, J. S., Li, Y., Valdesolo, P. & Kassam, K. S. Emotion and decision making. *Annu. Rev. Psychol.* **66**, 799–823 (2015). 25251484 10.1146/annurev-psych-010213-115043

[85] Mayer, J. D., Salovey, P. & Caruso, D. R. Emotional intelligence: Theory, findings, and implications. *Psychol. Inq.* **15**, 197–215 (2004).

[86] Mayer, J. D., Salovey, P., Caruso, D. R. & Sitarenios, G. Emotional Intelligence as a Standard Intelligence. *Emotion* **1**, 232–242 (2001). 12934682

[87] Bergström, T. et al. How do people talk decades later about their crisis that we call psychosis? A qualitative study of the personal meaning-making process. *Psychosis* **11**, 105–115 (2019).

[88] Fonagy, P. & Bateman, A. Progress in the treatment of borderline personality disorder. *Br. J. Psychiatry* **188**, 1–3 (2006). 16388061 10.1192/bjp.bp.105.012088

[89] Lysaker, P. H. & Klion, R. E. *Recovery, meaning-making, and severe mental illness: A comprehensive guide to metacognitive reflection and insight therapy. Recovery, meaning-making, and severe mental illness: A comprehensive guide to metacognitive reflection and insight therapy* (Routledge/Taylor & Francis Group, 2018).

[90] Butterfill, S. A. Interacting mindreaders. *Philos. Stud.* **165**, 841–863 (2013).

[91] CORCORAN, R. & FRITH, C. D. Autobiographical memory and theory of mind: evidence of a relationship in schizophrenia. *Psychol. Med.* **33**, 897–905 (2003). 12877404 10.1017/s0033291703007529

[92] Dimaggio, G., Lysaker, P. H., Carcione, A., Nicolò, G. & Semerari, A. Know yourself and you shall know the other… to a certain extent: Multiple paths of influence of self-reflection on mindreading. *Conscious. Cogn.* **17**, 778–789 (2008). 18394921 10.1016/j.concog.2008.02.005

[93] Böckler, A., Herrmann, L., Trautwein, F.-M., Holmes, T. & Singer, T. Know Thy Selves: Learning to Understand Oneself Increases the Ability to Understand Others. *J. Cogn. Enhanc.* **1**, 197–209 (2017). 32226919 10.1007/s41465-017-0023-6 PMC7089715

[94] Mehl, S., Rief, W., Mink, K., Lüllmann, E. & Lincoln, T. M. Social performance is more closely associated with theory of mind and autobiographical memory than with psychopathological symptoms in clinically stable patients with schizophrenia-spectrum disorders. *Psychiatry Res.* **178**, 276–283 (2010). 20494454 10.1016/j.psychres.2009.10.004

[95] Weijers, J. et al. Mentalization-based treatment for psychotic disorder: a rater-blinded, multi-center, randomized controlled trial. *Psychol. Med.* **51**, 2846–2855 (2021). 32466811 10.1017/S0033291720001506 PMC8640364

[96] Brent, B. K. & Fonagy, P. Chapter 15 - A mentalization-based treatment approach to disturbances of social understanding in schizophrenia. In (eds. Lysaker, P. H., Dimaggio, G. & Brüne, M.) Social cognition and metacognition in schizophrenia 245–259 (Academic Press, 2014). 10.1016/B978-0-12-405172-0.00015-6.

[97] Weijers, J. G. et al. Mentalization and Psychosis: A Rationale for the Use of Mentalization Theory to Understand and Treat Non-affective Psychotic Disorder. *J. Contemp. Psychother.* **50**, 223–232 (2020).

[98] Asen, E. & Fonagy, P. Mentalization-based Therapeutic Interventions for Families. *J. Fam. Ther.* **34**, 347–370 (2012).

[99] Gervinskaitė-Paulaitienė, L., Byrne, G. & Barkauskienė, R. Mentalization-Based Parenting Program for Child Maltreatment Prevention: A Pre–Post Study of 12-Week Lighthouse Group Program. *Children* **10**, 1047 (2023). 10.3390/children10061047 PMC10297588 37371278

[100] Byrne, G., Murphy, S. & Connon, G. Mentalization-based treatments with children and families: A systematic review of the literature. *Clin. Child Psychol. Psychiatry* **25**, 1022–1048 (2020). 32493055 10.1177/1359104520920689

[101] Kykyri, V.-L. et al. Soft Prosody and Embodied Attunement in Therapeutic Interaction: A Multimethod Case Study of a Moment of Change. *J. Constr. Psychol.* **30**, 211–234 (2017).

[102] De Sousa, P., Sellwood, W., Griffiths, M. & Bentall, R. P. Disorganisation, thought disorder and socio-cognitive functioning in schizophrenia spectrum disorders. *Br. J. Psychiatry* **214**, 103–112 (2019). 30139394 10.1192/bjp.2018.160

[103] Garety, P. A. & Freeman, D. The past and future of delusions research: from the inexplicable to the treatable. *Br. J. Psychiatry* **203**, 327–333 (2013). 24187067 10.1192/bjp.bp.113.126953

[104] Aaltonen, J., Seikkula, J. & Lehtinen, K. The comprehensive open-dialogue approach in western lapland: I. The incidence of non-affective psychosis and prodromal states. *Psychosis* **3**, 179–191 (2011).

[105] Luyten, P., Campbell, C., Allison, E. & Fonagy, P. The Mentalizing Approach to Psychopathology: State of the Art and Future Directions. *Annu. Rev. Clin. Psychol.* **16**, 297–325 (2020). 32023093 10.1146/annurev-clinpsy-071919-015355

[106] Fonagy, P. & Allison, E. The role of mentalizing and epistemic trust in the therapeutic relationship. *Psychotherapy* **51**, 372–380 (2014). 24773092 10.1037/a0036505

[107] Hogg, L. I. et al. A randomised feasibility trial comparing group and individual format GROUPS FOR HEALTH interventions for loneliness in people who experience psychosis. *Psychol. Psychother. Theory, Res. Pract*. 10.1111/papt.12574 (2025). 10.1111/papt.12574 PMC12065070 39878384

[108] Hendy, C., Tew, J. & Carr, S. Conceptualizing the peer contribution in Open Dialogue practice. *Front. Psychol.* **14**, 1176839 (2023). 37663329 10.3389/fpsyg.2023.1176839 PMC10469927

[109] Fedosejevs, V., Shi, J. & Hopfenbeck, M. S. Development of the peer-supported open dialogue attitude and competence inventory for practitioners: A Delphi study. *Front. Psychol.* **14**, 1059103 (2023). 37063576 10.3389/fpsyg.2023.1059103 PMC10097924

[110] Penn, D. L., Sanna, L. J. & Roberts, D. L. Social cognition in schizophrenia: An overview. *Schizophr. Bull.* **34**, 408–411 (2008). 18375928 10.1093/schbul/sbn014 PMC2632430

[111] Gervinskaitė-Paulaitienė, L., Ruggiero, M., Taubner, S., Volkert, J. & Barkauskienė, R. A follow-up study of the “Lighthouse” mentalization-based parenting program: Mentalization as a mediator of change. *Clin. Child Psychol. Psychiatry* **29**, 867–881 (2024). 38093217 10.1177/13591045231220965 PMC11188570

[112] Van Eck, R. M., Burger, T. J., Vellinga, A., Schirmbeck, F. & de Haan, L. The Relationship Between Clinical and Personal Recovery in Patients With Schizophrenia Spectrum Disorders: A Systematic Review and Meta-analysis. *Schizophr. Bull.* **44**, 631–642 (2018). 29036720 10.1093/schbul/sbx088 PMC5890469

[113] Seikkula, J. et al. Five-year experience of first-episode nonaffective psychosis in open-dialogue approach: Treatment principles, follow-up outcomes, and two case studies. *Psychother. Res.* **16**, 214–228 (2006).

[114] Leendertse, J. C. P. et al. Personal Recovery in People With a Psychotic Disorder: A Systematic Review and Meta-Analysis of Associated Factors. *Front. Psychiatry* **12**, 622628 (2021). 10.3389/fpsyt.2021.622628 PMC7940758 33708145

[115] Bergström, T. From treatment of mental disorders to the treatment of difficult life situations: A hypothesis and rationale - ScienceDirect. *Med. Hypotheses* **176**, 111099 (2023).

[116] Von Peter, S. et al. Open dialogue as a human rights-aligned approach. *Front. Psychiatry* **10**, 387 (2019). 10.3389/fpsyt.2019.00387 PMC6555154 31214063

[117] Patel, V. et al. Transforming mental health systems globally: principles and policy recommendations. *Lancet* **402**, 656–666 (2023). 37597892 10.1016/S0140-6736(23)00918-2

